# Change in paternal grandmothers´ early food supply influenced cardiovascular mortality of the female grandchildren

**DOI:** 10.1186/1471-2156-15-12

**Published:** 2014-02-20

**Authors:** Lars Olov Bygren, Petter Tinghög, John Carstensen, Sören Edvinsson, Gunnar Kaati, Marcus E Pembrey, Michael Sjöström

**Affiliations:** 1 Department of Biosciences and Nutrition, Karolinska Institutet, Huddinge, Sweden; 2 Department of Community Medicine and Rehabilitation, University of Umeå, Umeå, Sweden; 3 Department of Clinical Neuroscience, Karolinska Institutet, Solna, Sweden; 4 Department of Medical and Health Sciences, University of Linköping, Linköping, Sweden; 5 The Demographic Database, University of Umeå, Umeå, Sweden; 6 Institute of Child Health, University College London, London, UK

**Keywords:** Epidemiology, Food change, Environmental shock, Human transgenerational response, Cardiovascular mortality, Överkalix

## Abstract

**Background:**

This study investigated whether large fluctuations in food availability during grandparents' early development influenced grandchildren's cardiovascular mortality. We reported earlier that changes in availability of food - from good to poor or from poor to good - during intrauterine development was followed by a double risk of sudden death as an adult, and that mortality rate can be associated with ancestors´ childhood availability of food. We have now studied transgenerational responses (TGR) to sharp differences of harvest between two consecutive years´ for ancestors of 317 people in Överkalix, Sweden.

**Results:**

The confidence intervals were very wide but we found a striking TGR. There was no response in cardiovascular mortality in the grandchild from sharp changes of early exposure, experienced by three of the four grandparents (maternal grandparents and paternal grandfathers). If, however, the paternal grandmother up to puberty lived through a sharp change in food supply from one year to next, her sons´ daughters had an excess risk for cardiovascular mortality (HR 2.69, 95% confidence interval 1.05-6.92). Selection or learning and imitation are unlikely explanations. X-linked epigenetic inheritance via spermatozoa seemed to be plausible, with the transmission, limited to being through the father, possibly explained by the sex differences in meiosis.

**Conclusion:**

The shock of change in food availability seems to give specific transgenerational responses.

## Background

Early life exposure of humans to variable levels of availability of food leads to social, psychological, and biological responses that might be beneficial for health and survival in the short run but might also be detrimental in a longer perspective. Children raised in a poor area and poor circumstances, that changes to be more prosperous, suffer more often from myocardial infarction as adults [1]. Adults who as embryos or fetuses experience a change of environments, as a result of famine early or late in the intra-uterine period, often suffer from adult stroke [2]. Boys who are thin at birth but catch-up in weight often suffer from adult coronary heart disease as adults [3]. The question posed here is whether sharp and drastic changes, both in positive and negative direction, in the supply of food impact on cardiovascular mortality in descendants.

There is, in a parish in North Sweden, an opportunity to follow up a natural experiment and study how varying food-related circumstances influence health and survival short- and long-term. Such a study requires data from statistics on harvests and food prices and the local accounts of family histories. They were available in the setting of this study.

Food availability in northern Sweden during the 19th century depended largely on quality and size of harvests. After a poor harvest, the worst period was during early spring the following year. During much of the 19th century relief from southern Sweden was difficult at this time of the year as there was no rail service available and the frozen Baltic Sea prevented supplies to reach the north via ship. Conversely, after a good harvest, neighboring parishes did not need to purchase the surplus food and preserving it to next year was difficult. It was probably consumed creating large variations in food energy for the individual from year to year. In the first half of the 19th century sharp changes from one year to the next were not uncommon. It can be assumed that effects of food-related variations are not restricted to the exposed generation. The variations can influence future generations through mechanisms of learning and imitation, inheritance of goods and through biological processes. The latter have been studied in plant and animal research and transgenerational effects, most likely epigenetic inheritance, has been found [4-6]. A few epidemiological findings in humans indicate such a connection as well [7-11].

**Figure 1:**
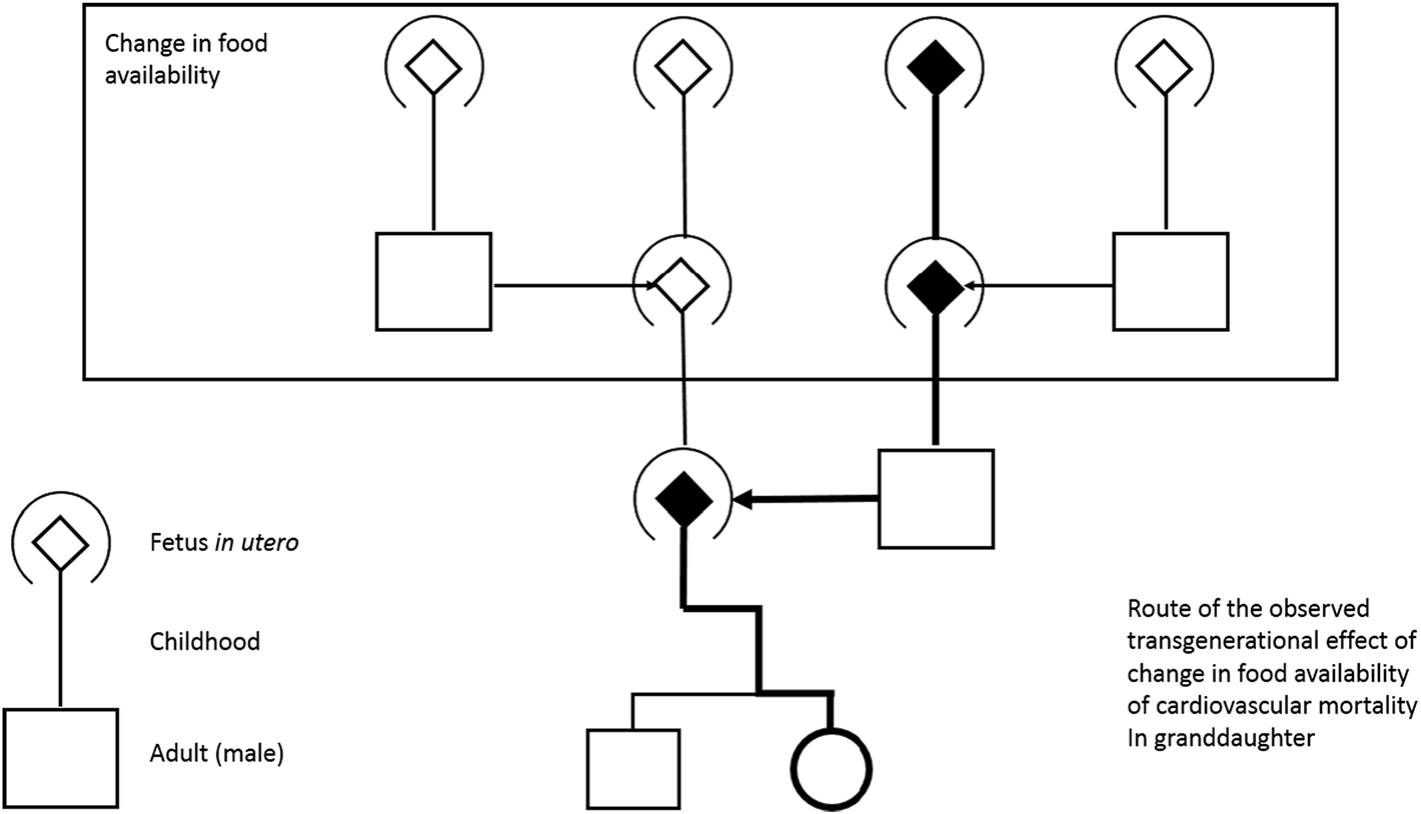
**A three-generation developmental pedigree that includes the fetus *in utero *and a childhood line linking fetus to adulthood.** The four grandparents of the male/female Överkalix probands are shown and the developmental stage in the grandparental early life when they were exposed to sharp changes in food availability. The broad line depicts the transmission route of the observed transgenerational response from paternal grandmother to granddaughter.

We studied sharp changes of food-related circumstances from one year to next and the influence on cardiovascular disease in families over three generations. Our analysis revealed a transgenerational response to sharp change, either to more or less food, in the early life environment. It was striking, that this response could only be demonstrated when the paternal grandmother had this experience, but none of the other grandparents (Figure 1.)

## Methods

The material, described elsewhere [8], consisted of 317 people randomly drawn from people born in 1890, 1905, and 1920 in Överkalix, Sweden. Pedigrees including more than 1200 parents and grandparents were obtained in the local parish registers in Sweden and Finland. Families where data were missing on birthdates, childhood circumstances or causes of death, were excluded from analyses. This left us with 277 families (87.4%) for the analysis.

### The ancestors´ nutrition

Assessment of the availability of food in the province was based on harvest and price statistics and the estimates of a 19th century statistician^2^. Food availability was classified as poor, moderate, or good. Harvests were usually completed in September and pigs and cattle were slaughtered later in the fall. November 1st was set to represent the best time following a good harvest. May 1st the subsequent year was set as the worst time following a crop failure. During the 19th century, people living in the study area experienced 20 years of poor availability of food, 20 years of good availability, and 60 years of moderate availability of food [7].

### Variables

The independent variables were sharp changes from one year to next in availability of food. We identified two occasions, 1812–13 and 1821–22, when crop failure one year was followed by good harvests the following summer. A grandparent exposed to this biennial change had his or her hardest time with respect to food availability in the spring following the crop failure the previous summer. For these grandparents, food availability improved at its height in the fall of the second year. In three instances, 1799–1800, 1876–77, and 1880–81, good harvests were followed by crop failure. The grandparents then had good availability of food peaking during the fall in the first year, but their hardship began with fall the second year and reached its height in spring the third year. The grandparents were classified as exposed or not exposed if they had or had not experienced any of these drastic changes in food availability during their early life, ranging from embryo to the attained age of 13 years of age. The exposure window was defined after our earlier epidemiological findings on transgenerational responses to ancestral exposure during this period (9) and was wide in order to increase the number of individuals exposed. The grandparents had either been exposed to a year of crop failure followed by a year of good harvest or a good harvest followed by a crop failure the next year. Other years the harvests could be good, bad, or intermediate, which was considered in a sensitive analysis. Given the exposure window was large, we also compared the effects of exposures from 0–5 and from 6-12 years. The outcome was defined as mortality in diseases of Chapter VII, Cardiovascular disease, the International Classification of Diseases, Ninth Revision (ICD9) [12], registered as direct, intermediate, underlying or contributing cause of death. Individual covariates were birth year (1890, 1905, or 1920), father's occupancy in two classes (laborer and having no farmland versus other occupancies), parents' death during the period, parents' literacy as ascertained by the clergy, and gender (by stratification).

### Ethical considerations

The personal integrity loss has been found to be balanced by the possible importance of the findings and the study was approved by the Regional Ethical Committee of North Sweden, Medicine (Dnr 06-046 M).

### Statistical analysis

Proportional Hazards Model was applied following the index persons up to death or December 31st 2011. The analyzes were conducted to investigate whether an ancestor's sharp change in food availability predicted cardiovascular disease as a cause of death of the index person. Separate analyzes were also performed for sharp food availability changes that went from poor to good and from good to poor. All analyzes were stratified by gender. Possible confounding effects from another six factors were considered: birth-cohort, father's occupancy, parent´s deaths before the subject turned 13, and parents' literacy. A covariate was included in the models if the log-rank test revealed that it was associated with cardiovascular disease (p < 0.25). The proportionality assumption was assessed by including the interaction term between the predictors and time. These computations showed that none of the predictor variables in any of the presented models interacted significantly with time (p > 0.05), indicating that the proportionality assumption was not violated.

Potential consanguinity bias was assessed by randomly excluding multiple cousins and rerunning the analyzes. Moreover, sensitivity analyzes examined possible confounding influences of food availability levels, other than the sharp changes of supply, experienced by the ancestors during their childhood or intrauterine development. To accomplish this, new variables were constructed in which maternal and paternal grandfathers and grandmothers were classified by number of such exposures: 0–2 times, 3–4 times, or more than 4 times. The eight new variables (two variables for every type of grandparent) were entered in a backward stepwise manner into the models with the exclusion criteria set at p > 0.25, keeping the factors included in the original analyzes (see Table 1 or 2) as forced variables. Belonging to one of the three cohorts were adjusted for as there was an increase in life span between the first (1890) and the last (1920) birth cohort. The data analyzes were generated using the IBM SPSS statics, 18.0 (SPSS, Inc., Chicago, Illinois).

**Table 1:** Grandparents' childhood experience of drastic change in food availability, from one year to the next year, by descendants' cardiovascular mortality*

	**Men**	**Women**
Paternal grandfather	0.87 (0.46-1.64)	0.91 (0.43-1.96)
Paternal grandmother	0.64 (0.32-1.29)	**2.69 (1.05-6.92**)
Maternal grandfather	1.26 (0.68-2.34)	1.32 (0.58-3.04)
Maternal grandmother	0.69 (0.35-1.36)	0.56 (0.22-1.49)
Index cases	151	126

*Associations are presented as Hazards ratios (HRs) with 95% confidence intervals (CIs 95%). The models are adjusted for birth cohort, mother´s literacy, father´s death before the index person attained 13 years of age. Bold numbers indicate significant associations, p<0.05.

**Table 2:** Grandparents' childhood experience of drastic change in food availability, from one year to the next year, going from poor to good and good to poor by descendants' cardiovascular mortality*

	**Poor to good**	**Poor to good**	**Good to poor**	**Good to poor**
	Men	Women	Men	Women
Paternal grandfather	0.90 (0.45-1.80)	1.42 (0.64-3.14)	1.08 (0.29-4.00)	0.15 (0.02-1.41)
Patermal grandmother	0.54 (0.24-1.20)	1.62 (0.58-4.52)	0.86 (0.22-3.3)	**21.27 (2.24-202.47)**
Maternal grandfather	1.17 (0.58-2.35)	1.22 (0.46-3.23)	1.28 (0.31-5.34)	3.37 (0.59-19.22)
Maternal Grandmother	0.60 (0.24-1.49)	0.91 (0.30-2.75)	0.75 (0.22-2.58)	0.16 (0.02-1.32)
Index cases	151	151	126	126

*Associations are presented as Hazards ratios (HRs) with 95% confidence intervals (CIs 95%). The models are adjusted for birth cohort, mother´s literacy, father´s death before the index person attained 13 years of age. Bold numbers indicate significant associations, p<0.05.

## Results and discussion

When/if a paternal grandmother experienced drastic changes, from good to poor and from poor to good, of food availability as a child, then her granddaughters had an increased Hazards Ratio (HR) for cardiovascular mortality as an adult (HR 2.69, CI 95% 1.05-6.92). Such an effect from childhood experience of the grandfathers or maternal grandmothers, i.e. the three other grandparents was not observable (Table 1, Figure 1). In the consanguinity bias test, first cousins considered, the association between paternal grandmothers and granddaughters increased even further (HR 5.48, CI 95% 1.75-17.26), but the other associations between type of grandparent and descendants remained non-significant with similar HRs. Sensitivity analyses with adjustments for grandparents' numbers of years of exposure to poor and good food availability in early development revealed no marked differences (Data not shown), as expected after our earlier findings on the difference in responses to persistency and change of exposure (2). The exposure window encompassing the early life up to puberty was halved to check for effects. No marked differences between the two halves were detected as to paternal grandmothers´ influence on the transgenerational responses. The HR was around 2 for both and the p-values for these estimates were 0.06 and 0.10.

Female descendants whose paternal grandmothers experienced a year with good harvest directly followed by a year of crop failure had an increased risk of death by cardiovascular disease (HR 21.27, CI 95% 2.24-202.47). This association also remained significant (p < 0.05) after adjusting for the number of crop failures and good harvests experienced by grandparents in their early development. Due to a low number of females in our sample that actually had grandparents who had experienced this type of sharp change in supply, the test for ruling out consanguinity bias could not be conducted.

The opposite direction of sharp change from one year to next, from poor to good food availability, experienced by the paternal grandmother predicted excess risk, although non-significant, of cardiovascular deaths among granddaughters (HR 1.62, CI 95% 0.58-4.52). Accounting for the grandparents' number of crop failures or good harvests experienced during their early development, however, revealed a significant level (HR 4.45, p = 0.02). The association was further corroborated by the fact that the analysis where cousins were excluded from the sample provided a similar range of Hazards Ratio (HR 3.73, CI 95% 1.10-12.59).

Only a connection between the paternal grandmother and her son´s daughter could be demonstrated (Table 2). Neither of the two directions of sharp changes in food availability experienced by the paternal grandfather, the maternal grandfather or the maternal grandmother, was significantly associated with cardiovascular mortality. In these cases, the associations remained non-significant (p > 0.05) when the two described sensitivity tests were carried out. Our analysis revealed a transgenerational response to sharp change, either to more or less food, in the early life environment. The confidence intervals were very large but it is intriguing that with these early life experiences for three of the four grandparents (maternal grandparents and paternal grandfather), no influence on grandchild’s' cardiovascular mortality could be seen. Only when paternal grandmothers´ food supply had these changes was it followed by high cardiovascular grandchild mortality, and then only by the sons´ daughters, not the sons´ sons (p = 0.016). How do we explain these rather peculiar phenomena?

Earlier we have shown other transgenerational response transmission routes of influence, going for instance from paternal grandfather to the son´s son. In general, epigenetic inheritance in the broadest sense (i.e. including microRNAs) via the gametes is a plausible candidate for explaining the TGRs in mammals [4,13]. This is particularly so for paternal transmission, since maternal transmission can also be via the oval cytoplasm (including mitochondria) or transplacental metabolic signals. The molecular mechanisms mediating the causal link between exposure and descendant outcome, particularly the TGR signal, are unclear and could involve DNA methylation, DNA change at a genotoxic sensor, change in telomere length, viruses, prions, RNA molecules, or transcript fragments carried by the spermatozoon [14-16]. Whatever the mechanisms, acquired DNA methylations sometimes survive the early eradication and reinstitution after fertilization and reappear in descendants [13,17-20]. Our results in humans could indicate similar molecular transgenerational findings as those found in mice [4,21]. Relevant to our findings is Dunn and Bale’s study of maternal high-fat diet exposure in mice that showed transgenerational effects on body size and insulin sensitivity down to the F3 generation. Interestingly, they found that only F3 females displayed the increased body size phenotype and this effect was only passed via the paternal lineage [4].

Why is it that only the paternal grandmothers's son's daughter show a TGR and no TGR is induced via the other three grandparents could be demonstrated? This particular route of transmission, from grandmother to her son´s daughter, suggests the TGR signal it carried on the X chromosome. The father receives his only X from his mother and only passes it on to his daughters. But if the X chromosome in father’s germ-line cells is able to acquire an epigenetic mark as a result of transmitted metabolic changes consequent upon his mother’s response to early life sudden food change , why don't we see a TGR in the maternal grandmother's daughter's children? There may be general reasons for this related to the intergenerational flow of metabolic signals, via the egg cytoplasm or across the placenta, that mask any specific epigeneticTGR. Indeed, it has been proposed that there is an integrated nutritional signal down the female line that counteracts short term TGRs [22].

There may also be a specific explanation if the TGR signal is indeed epigenetic mark(s) on the germ-line X chromosome. When the middle generation person is a woman the two X's will pair and recombine during meiosis which is likely to erase the epigenetic marks that constitute the TGR signal. If the middle generation is a man, nearly all his X and Y do not pair or recombine, thus increasing the possibility that any epigenetic signal remains intact. This signaling could have modified many gene expressions of significance for cardiovascular disease either indirectly through changes in X-linked microRNAs affecting many gene targets elsewhere or directly changing expression of X-linked genes relevant to cardiovascular pathology. One such gene is an X-linked member of the family of Inhibitor Apoptopic Proteins (XIAP) involved in myocardial cell survival in the face of ischemia [23,24].

In epidemiology, and to some extent today in molecular biology, especially in the field of epigenetics, unpredictable environmental change is often considered to be an important agent in the etiology of diseases [25]. We found earlier that change of nutrition during fetal development doubled the risk of adult stroke intragenerational mortality [2] leading to considerations of whether transgenerational responses might followed the same pattern. Our transgenerational results, whilst providing no direct epigenetic evidence, do lend support to the hypothesis that gametic epigenetic inheritance might explain the paternal transmission within our epidemiological findings.

To our earlier findings, we now add the possibility that the first shock of starvation and stress and a sharp change to plenty, are peak moments in inducing gene expression responses and epigenetic processes. To merge in analysis these peaks with very long periods of famine could, for example, explain an absence of influence on later mortality seen in a two-year crop failure found in Finland [26].

The strength of this study is the natural experiment exposing humans to large variations in food supply. The food availability depended on the weather, which struck haphazardly. Poor and good harvest affected the whole community. Following a poor harvest, most likely all families had little to eat. The social stratification of the grandparental generation was flat with very few wealthy persons. Food relief from other parts of Sweden was not possible over the frozen Baltic Sea and in the absence of railway. Not only failures of crop contributed to the variation. After very good harvests, which were as common as crop failures, the food could not be sold with reasonable profit, long-term conserving was limited over the summer the following year, and the extra abundance of food probably was consumed before summer.

The sample size was however small and confidence intervals large. The exposure to food availability is in part a measure of nutrition status, but the whole society was put under a hardship and mechanisms other than nutritional could have been at work. The outcome is measured with the cardiovascular mortality of the grandchildren. Their nutrition during early and later life could have had an effect on their adult cardiovascular mortality, but they were not exposed to any large fluctuations in crops. Social environment, childhood experiences, and psychological circumstances [27] might have a long-term influence on the probands' mortality rate. To control for these effects, the probands' early life circumstances were adjusted for in the analyses. The life expectancy for the three birth cohorts were different, longer for the later cohorts and hence belonging to cohort was adjusted for. In the 19th and 18th century, the research area was isolated and very homogeneous in terms of population. In such populations, recessive monogenetic diseases may emerge due to a small founder population and subsequent inbreeding. The in-mating and small number or founders since the medieval time however, probably has diminished the variation in hard-wired genetics ,which should facilitate the detection of gametic epigenetic inheritance. Selection in this material, measured as diminishing variance of longevity or reproductive fitness, has been ruled out elsewhere as an explanation for the other trans-generational responses to food availability found in this population [8].

## Conclusion

Our results raise a suggestion that studies of the effects of environmental induction of transgenerational response consider the change of exposure, in both directions, in addition to the adverse or beneficial doses. Insight into the character, epigenetic or otherwise, of these responses might lead to new disease preventions and treatments and avoidance of inadvertent harm from va too sudden, short term intervention, having as side effect the disease agent of this study, change of availability of food.

## Competing interests

The authors declare no conflicts of interests.

## Authors´ contributions

LOB contributed with design, acquisition of data, and text, PT with analysis and text, JC with analysis, critical review and text, SE with design, historical analysis and text, GK with design, acquisition of data and text, MP with design, critical review, epigenetic theory, and text, MS with design, critical review, and text. All have made substantial contributions to conception and design or analysis and interpretation of data. All have been involved in the drafting of the manuscript and revising it for important intellectual content. All authors read and approved the final manuscript.
